# The Ischemic Environment Drives Microglia and Macrophage Function

**DOI:** 10.3389/fneur.2015.00081

**Published:** 2015-04-08

**Authors:** Stefano Fumagalli, Carlo Perego, Francesca Pischiutta, Elisa R. Zanier, Maria-Grazia De Simoni

**Affiliations:** ^1^Department of Neuroscience, IRCCS-Istituto di Ricerche Farmacologiche Mario Negri, Milan, Italy; ^2^Department of Pathophysiology and Transplantation, Fondazione IRCCS Ca’ Granda-Ospedale Maggiore Policlinico, Milan, Italy

**Keywords:** neuroinflammation, microglia, macrophages, acute brain injury, phenotypical polarization, cell morphology

## Abstract

Cells of myeloid origin, such as microglia and macrophages, act at the crossroads of several inflammatory mechanisms during pathophysiology. Besides pro-inflammatory activity (M1 polarization), myeloid cells acquire protective functions (M2) and participate in the neuroprotective innate mechanisms after brain injury. Experimental research is making considerable efforts to understand the rules that regulate the balance between toxic and protective brain innate immunity. Environmental changes affect microglia/macrophage functions. Hypoxia can affect myeloid cell distribution, activity, and phenotype. With their intrinsic differences, microglia and macrophages respond differently to hypoxia, the former depending on ATP to activate and the latter switching to anaerobic metabolism and adapting to hypoxia. Myeloid cell functions include homeostasis control, damage-sensing activity, chemotaxis, and phagocytosis, all distinctive features of these cells. Specific markers and morphologies enable to recognize each functional state. To ensure homeostasis and activate when needed, microglia/macrophage physiology is finely tuned. Microglia are controlled by several neuron-derived components, including contact-dependent inhibitory signals and soluble molecules. Changes in this control can cause chronic activation or priming with specific functional consequences. Strategies, such as stem cell treatment, may enhance microglia protective polarization. This review presents data from the literature that has greatly advanced our understanding of myeloid cell action in brain injury. We discuss the selective responses of microglia and macrophages to hypoxia after stroke and review relevant markers with the aim of defining the different subpopulations of myeloid cells that are recruited to the injured site. We also cover the functional consequences of chronically active microglia and review pivotal works on microglia regulation that offer new therapeutic possibilities for acute brain injury.

## Classical View of Neuroinflammation

In the late 19th century, Paul Ehrlich observed that a water-soluble viable dye injected into the peripheral circulation stained all organs except the central nervous system (CNS), providing the first indication that the CNS was anatomically separated from the rest of the body. The idea that the brain was a unique anatomical compartment was further confirmed by Edwin Goldman who showed that a dye injected into the spinal fluid did not stain peripheral tissues. This is of course due to the blood–brain barrier (BBB) that restricts access of soluble factors to the brain, including 98% of antibodies and immune cells. This feature together with the lack of a lymphatic system, low constitutive levels of major histocompatibility complex (MHC) class I and II molecules, local production of suppressive factors, and limited numbers of professional antigen-presenting cells drove the concept of the CNS as an immuno-privileged site (1).

In normal conditions, the presence and trafficking of immune cells in the brain are negligible. However, we now know that the brain is far from being an inactive immune organ. It has actually its own resident immune population, microglia, in addition to the fact that the BBB can allow active import of immune molecules and cells. After injury, brain immunity includes a variety of events that develop non-linearly in response to multiple factors and involve complex interactions between cells and environmental signals. As a consequence, in CNS disorders, activation of distinct inflammatory pathways may affect the course of an injury in different and possibly opposing ways (2).

Cells of myeloid origin, such as microglia and macrophages, are major actors in brain inflammation, a hallmark of acute brain injury. Microglia reside in the CNS and actively monitor the surrounding microenvironment (3). Under physiological conditions, these cells play a variety of roles that go beyond classical inflammatory activity, including support to synaptic wiring during development and monitoring of neuronal firing in the mature brain, thus contributing to brain homeostasis (4, 5). After an acute CNS injury, microglia act mainly as a player of the immune system. Virtually all CNS disorders involve reactive microglia and the progression and resolution of many diseases also depend on microglial activity (6).

Microglia share the theater of their action with blood-borne macrophages, which infiltrate into the inflamed CNS. Both cell populations show a range of functional states, with a specific pattern of receptor expression, molecule production, and morphological feature acquisition. The inflamed environment plays a major role in the definition of their overall function by providing stimuli that induce specific phenotypical/functional states.

An open challenge is to properly characterize the roles of microglia and macrophages in brain injury progression and resolution. This would help define the milestones for effective manipulation of brain inflammation, with the specific aim of favoring its protective arm and boosting innate neuroprotective mechanisms. This review will look at the latest findings on inflammation in acute brain injury, specifically addressing the impact of environmental signals on the function of microglia and macrophages in injury and neuroprotection. We will specifically discuss (1) the latest view of neuroinflammatory mechanisms, depicting the rationale for focusing on myeloid cell protective modulation; (2) the ability of the inflamed environment to drive myeloid cell behavior, particularly their distribution, expression markers, and morphology; (3) the impact of selectively primed/modulated states of microglia on the exacerbation or resolution of neurological disorders.

## Changing the Angle: A New View of Neuroinflammation

Data accumulated over the last decade have deeply changed the general view of inflammation in the CNS. A striking new concept has been that brain immunity, in addition to the well-known pro-inflammatory actions, can initiate protective mechanisms by exploiting the bivalent nature of microglia and macrophages. *In vitro* experiments indicate that these cells may develop either a classic pro-inflammatory M1 or an alternative anti-inflammatory and pro-healing M2 polarization (7). *In vivo*, microglia and macrophages appear to acquire intermediate phenotypes, whose ultimate functions rely on the combination of different polarization markers ranging from M1 to M2 (2, 8). Multiple factors concur to determine myeloid cell functional states and may offer a way to therapeutically manipulate myeloid cell activation. From this viewpoint, microglia and macrophages seem at the crossroads of several inflammatory mechanisms, throughout the entire course of brain pathophysiological events. They can be viewed as *in situ* expert operators whose timely actions may contain and resolve brain injury. Experimental research is now dedicating considerable effort to understanding the rules that govern brain innate immunity, to implement strategies for manipulating the innate immune response to favor its protective functions (9).

In the inflamed CNS, activated microglia and recruited macrophages present some common features and some distinct characteristics. Common features include the expression of common phenotypic markers, the ability to polarize toward M1/M2 phenotypes, the phagocytic behavior, and the ameboid shape that activated microglia may acquire. Microglia and macrophages, however, differ in several aspects and recent work has attributed exclusive features to each of these cell populations. Microglia originate from primitive hematopoiesis in the fetal yolk sac, take up residence in the brain during early fetal development, and retain the ability to proliferate. By contrast, macrophages derive from granulocyte–monocyte progenitors during both development and adulthood (6, 10). Microglia have a lower turnover rate than macrophages: respectively 6 months and 17 h in mice (11). The activation of microglia depends on ATP/ADP signaling, whereas macrophages are equipped to maintain viability and function in hypoxia/ATP loss (3, 10). Finally, only microglia have a ramified morphology, with branches that emerge from the cell body and communicate with surrounding neurons and other glial cells (12, 13).

Whether these differences imply different roles in brain injury progression and repair has yet to be fully determined, though there is increasing evidence that microglia should be considered functionally distinct from macrophages (14, 15).

## The Hypoxic Environment, A Major Cue to Microglia and Macrophage Activation

After acute brain injury such as stroke, traumatic brain injury (TBI), subarachnoid, or intracerebral hemorrhage, a series of neurochemical processes is unleashed and gives rise to a complex pathophysiological cascade that can be viewed as cellular bioenergetic failure triggered by hypoperfusion (16). Hypoperfusion leads to hypoxia, which precedes and causes detrimental events, such as excitotoxicity, oxidative stress, BBB dysfunction, microvascular injury, hemostatic activation, post-ischemic inflammation and, finally, cell death (17, 18). All these events contribute to changing the ischemic environment over time and, consequently, the behavior of microglia and macrophages. Because hypoxia immediately follows hypoperfusion, it affects the brain myeloid cell response to injury early. Here, we discuss the effects of hypoxia on microglia and macrophage behavior and the different activations and recruitments of these two populations in an ischemic environment.

### Microglia and macrophage behavior in hypoxic conditions

Microglia consume energy in an ATP-dependent manner for their broad range of activities, including inflammatory mediator production (19) and cytoskeleton reorganization (20, 21). Microglia are thus highly susceptible to energy deficits and local changes in blood perfusion after acute injury probably affects microglia reactivity and survival.

Hypoxia induces a time-dependent autophagic cell death in microglia cultures with increased release of pro-inflammatory cytokines (IL-8 and TNFα) through the hypoxia-inducible factor-1α (HIF-1α) dependent pathway (22). HIF-1α is a transcription factor responsible for the adaptation of cells to low oxygen tension, for the regulation of glucose metabolism and for cell proliferation and survival (23, 24). Its expression is induced in the ischemic brain (25). However, its exact role still needs clarification, as it has been seen to display either protective or detrimental functions (26–29).

Unlike microglia, macrophages can switch their metabolism to anaerobiosis and remain viable in hypoxic/ischemic conditions (10, 30). Many pathological processes, such as tumors, atherosclerosis, and ischemia, involve a low oxygen concentration and the concomitant presence of macrophages (31). Efforts have been made to clarify how macrophages adapt to low oxygen concentrations. The HIF-1α and nuclear factor κB (NF-κB) families of transcription factors are major regulators of this adaptation (31, 32). Myeloid cell-mediated inflammatory response requires HIF-1α and involves a decrease in the expression of inducible nitric oxide synthase (iNOS) and reduced production of ATP by glycolysis (33, 34). Hypoxia can mediate NF-κB activation, favoring the production of inflammatory cytokines (32), and hypoxia-induced CXCL12 expression regulates mobilization and homing of hematopoietic stem and progenitor cells to the ischemic tissue (35–37).

### Microglia and macrophage behavior in the ischemic brain lesion

The ischemic environment drives macrophage recruitment, and this results in the co-presence of infiltrating blood-borne macrophages and resident reactive microglia in the lesioned site (12). In experimental brain ischemia/reperfusion injury, green fluorescent protein (GFP)-expressing microglia studied by *in vivo* two-photon microscopy show prolonged resilience to ischemic conditions by becoming stalled, with reduced dynamic behavior. As blood perfusion is re-established, microglia recover their behavior and rearrange the cytoskeleton, acquiring either a bushy morphology, with multiple short processes around enlarged cell bodies (38, 39), or a reactive ameboid shape (21, 40).

When there is no reperfusion, e.g., after permanent ischemia or TBI, hypoxia may persist beyond the resilience limit of microglia, causing microglia irreversible damage and death (3). Accordingly, compared to ischemia/reperfusion, injuries with no reperfusion cause a larger lesion area depleted of microglia. The microglia-empty territory is rapidly replenished by round-shaped CX3CR1-/CD11b+/CD45^high^+ cells, which are likely to be the infiltrating macrophage population (8, 41).

**Figure 1:**
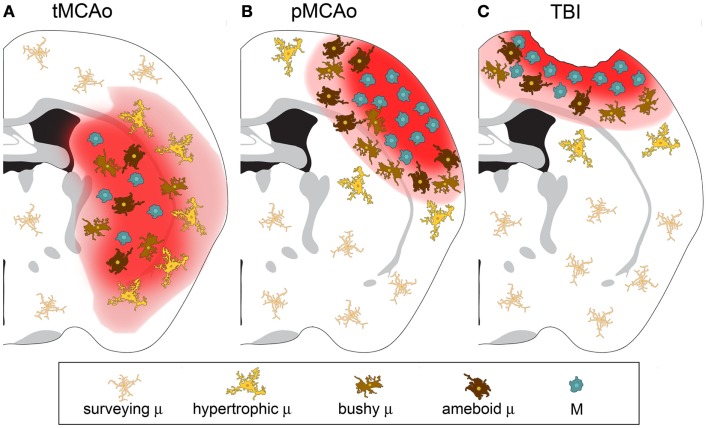
**Microglia and macrophage distribution over the lesion area 24 h after injury in three different models of acute brain injury**. **(A)** After transient middle cerebral artery occlusion (tMCAo), the lesion core is populated by infiltrated macrophages (M) and bushy/ameboid microglia (μ). The penumbra contains hypertrophic μ. **(B)** After permanent middle cerebral artery occlusion (pMCAo) no μ cells are present in the lesion core where there is high M infiltration. Bushy μ cells surround the lesion core. **(C)** The distribution is similar after traumatic brain injury (TBI). M are recruited to the lesioned tissue close to its lesion edge, while bushy and hypertrophic μ cells populate more distant areas.

As discussed above, macrophages can switch to an anaerobic metabolism and adapt to hypoxic/ischemic conditions. In experimental stroke models, immune cell infiltration is more evident after permanent than transient occlusion of the middle cerebral artery (MCA) (42, 43). A schematic representation of differential distribution of microglia and macrophages at early stages after permanent or transient ischemia or TBI is depicted in Figure 1.

The metabolic status of the lesioned environment is thus a major determinant in the recruitment/activation of myeloid cells in the CNS and in the balance between microglia and macrophages. The consequences of the specific composition of the myeloid population still need clarification. Yamasaki et al. recently compared the transcription profiles of microglia or monocyte-derived macrophages in a model of experimental autoimmune encephalopathy. They showed that microglia downregulated metabolic pathways, whereas macrophages displayed active phagocytic and pro-inflammatory behavior (44). Although this observation cannot be extended to other injury models, these findings reinforce the idea that microglia and macrophages have different intrinsic properties that govern their specific responses to environmental signals.

Macrophages engrafted microglia-depleted brain regions in *CD11b-HSVTK* mice (45), a model of selective microglia depletion obtained by intracerebroventricular valganciclovir (46, 47). In this model, macrophages infiltrated within 2 weeks after depletion and showed microglia-like behavior, extending processes toward an ATP source. This might indicate that macrophages could populate the adult brain and replace microglia in sustain cerebral homeostasis (45). After a severe acute injury with impairment of the cerebral blood flow and no reperfusion, brain homeostasis is disrupted and metabolic crisis occurs, leading to massive death of cerebral cells, including microglia. Macrophages invade these lesioned regions and become activated. In these conditions, their role is likely to be different from that of surveying microglia. At early times, infiltrated round-shaped CD11b/CD45^high^ cells express M2 polarization markers, while microglia are mostly located at lesion boundaries with lower expression of polarization markers (8).

The picture in acute phase of brain injury may not apply at longer times when other events, such as microglial proliferation, might define a different balance between microglia and macrophages. Microglia proliferate starting 72 h after focal brain ischemia induced with the filament model in mice (48). Microglial proliferation in the lesioned brain areas is affected by the severity of injury, being clearly observable after 30 min of transient ischemia, but only weakly after more severe 60 min of ischemia. This latter caused wide areas of microglia loss where subsequent replenishment with fresh microglia was limited (48).

Local proliferation may also be promoted by pericytes, as recently shown after permanent ischemia (49). In response to ischemia, pericytes may leave the vessel wall and settle in the brain parenchyma. Pericyte infiltration occurs specifically in the lesioned area depleted of microglia cells, reaches its peak 7 days after injury and is still detectable at 21 days. These infiltrated pericytes express typical microglia markers, such as Iba1, CD11b, and GAL-3, but interestingly, they are negative for CD68 and CD45^high^, these latter associated with leukocytes (49). Thus pericytes may function as a local source of microglia, and this may be a potential mechanism of microglial repopulation of severely injured areas.

The ischemic milieu changes over time, possibly also as a consequence of changes in blood perfusion. Areas with preserved blood flow soon after ischemia may have defective perfusion at later times because of the formation of secondary clots. This is generally referred to as “no-reflow” (50, 51) and may depend on different mechanisms, such as fibrin deposition and platelet activation (52–54), or clotting of immune cells in small capillaries (55). Delayed perfusion deficits can potentially affect microglial activity and proliferative ability in selected brain areas and shifting the balance between resident microglia and recruited macrophages.

## Surface Antigens: The Interface between Myeloid Cells and Environment

The differential behavior of microglia and macrophages in response to the hypoxic environment suggests intrinsic differences in the nature of these populations. However, in experimental research, microglia and macrophages are often referred to as an unique population, because their study relies on labeling non-selective surface antigens, which are expressed constitutively or after activation by either cell types. For some markers different patterns of expression may help distinguish resident from infiltrated myeloid cells. Here, we discuss the most widely used markers, particularly murine markers of myeloid cells, providing information on their interaction with the environment and on the biology of myeloid cell subtypes.

### Constitutive markers

**Figure 2:**
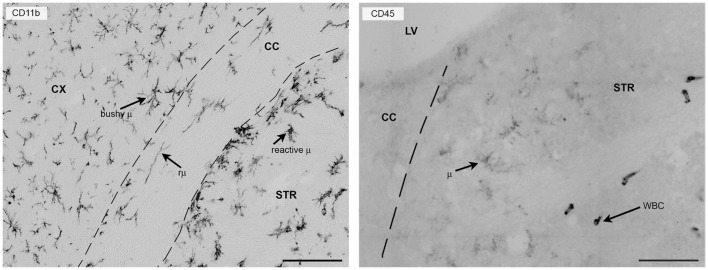
**CD11b or CD45 label myeloid cells in the mouse brain 24 h after focal transient ischemia**. Left panel: CD11b is commonly used to label myeloid cells in the mouse brain and provides detailed information on morphology because it is expressed uniformly on the cell membrane. Twenty-four hours after focal transient ischemia, different microglia cell types can be found in the hemisphere ipsi-lateral to the lesion. Bushy microglia (μ) populate the cortex (penumbra) and rod microglia (rμ) with elongated morphology are present in the white matter (corpus callosum) and hypertrophic reactive microglia (reactive μ) populate the striatum, the area that corresponds to the lesion core. Right panel: CD45 labeling helps distinguish resident microglia from recruited leukocytes. Microglia have ramified morphology and pale staining (CD45^low^), whereas infiltrated leukocytes (white blood cells, WBC) can be identified by their round-shaped morphology and strong, well-contrasted staining (CD45^high^). The elongated shape of CD45^high^ cells may depend on crawling along vessel walls or their perivascular location. Bars = 100 μm, microphotograms modified from Ref. (40). Neuro-anatomical structures are indicated by CX (cortex), STR (striatum), CC (corpus callosum), and LV (lateral ventricle).

CD11b is one of the most commonly used surface markers for immunostaining microglia/macrophages (8, 56). It belongs to the integrin family of surface receptors and is covalently bound to a β2 subunit to form integrin aMb2 (Mac-1, CD11b/CD18), which is implicated in diverse responses including cell-mediated killing, phagocytosis, chemotaxis, and cellular activation. CD11b is upregulated after microglia/macrophage activation and recognizes several ligands including C3 fragments, resulting from complement activation, fibrinogen, intercellular adhesion molecule-1 (ICAM-1), denatured products, and blood coagulation factor X. With its presence on the membrane surface and its constitutive expression, CD11b is particularly suitable for studying myeloid cell morphology in either physiological or pathological conditions (Figure 2).

Myeloid cells are often labeled by another constitutive marker, ionized calcium-binding adaptor 1 (Iba1) that provides information on their activation and morphology comparable to that with CD11b (57, 58). Iba1 is a calcium-binding protein specific for myeloid cells and has a role in bundling actin, in membrane ruffling and phagocytosis (59).

CD45 is a transmembrane glycoprotein expressed by cells of hematopoietic origin, except erythrocytes. It is a member of the protein tyrosine phosphatase (PTP) family. Its intracellular carboxy-terminal region contains two PTP catalytic domains, whereas the extracellular region varies widely due to alternative splicing of exons 4, 5, and 6 (designated as A, B, and C, respectively). CD45 has a role in T- and B-cell signal transduction and in the adult mouse the expression of selective isoforms depends on the cell type and activation state. Microglia express low constitutive levels of CD45 and even after activation they maintain lower levels of CD45 than circulating/infiltrating leukocytes. CD45 labeling, therefore, gives a weak signal in microglia that can be exploited to distinguish resident microglia from infiltrated immune cells by either immunohistochemistry or flowmetry (8, 40, 60, 61) (Figure 2).

Isolectin B4 binds α-d-galactosyl residues, which are expressed on microglia surface and on activated endothelial cells. This marker labels myeloid cells non-homogeneously and it also binds to endothelial cells (62). So it cannot be used to label microglia/macrophages clearly in tissue specimens. Thus, isolectin B4 is mainly employed to label *in vitro* microglial cells, giving a homogeneous staining and a quick immunostaining protocol (63, 64).

The receptor for fractalkine (CX3CR1) is constitutively present on microglia and is inducible in specific subsets of macrophages. Its main function in the brain is to support the communication between microglia and neurons. We discuss below the role of CX3CR1 and its deletion in different pathological conditions. Transgenic mice expressing GFP under the promoter of *cx3cr1* (*cx3cr1*^GFP/+^) are commonly used to visualize microglia in *in vivo* (40) and in *ex vivo* experimental settings (65). Our group has shown that the expression of GFP in *cx3cr1*^GFP/+^ mice helps distinguish microglia and infiltrated immune cells at early times (24 h) after ischemia (40) or TBI (41). Only ramified CD11b positive cells express GFP, whereas round-shaped cells, positive for CD11b and CD45^high^, or CD3 (a marker for lymphocytes) do not. The first subset of infiltrating macrophages is recruited through CCR receptors and they do not express CX3CR1. Circulating monocytes have been divided into two subsets, the classical one expressing the CCR family receptors and prone to tissue invasion upon recruitment (66–68) and the non-classical subset, positive for CX3CR1. This latter subset patrols along blood vessel walls and accumulates in peripheral tissues, such as spleen, lung, and liver (68–70). Non-classical monocytes are recruited in the brain at later stages after acute injury, so they are not present at early times, so CX3CR1 positivity can be used as a selective marker for resident microglia early after injury.

The identification of a unique microglial molecular fingerprint would facilitate the study of microglia biology and help develop their therapeutic modulation. Recent gene profiling of murine CD11b+/CD45^low^ microglia compared to that of Ly6C^high^ or Ly6C^low^ macrophages showed that there were a number of genes highly or uniquely expressed by microglia, such as *P2ry12*, *Gpr34*, *Mertk*, *C1qa*, *Gas6*, and *Fcrls* (71). FACS analysis indicated that antibodies against P2ry12 or FCRLS stained adult microglia, but not CD11b-gated myeloid cells isolated from spleen, bone marrow, and peripheral blood (71). P2ry12 and FCRLS need further validation, for instance in brain pathologies, but they offer promise as selective molecular markers for resident microglia.

The genetic profiles of microglia and macrophages have been recently associated with environmental signals that drive these cell identities (72). Gene profiling of mouse microglia and peritoneal macrophages revealed that the cerebral and peritoneal environments drive different programs of gene expression by differentially activating a common repertoire of enhancers. The activated enhancer repertoire in turn promotes the expression of secondary transcription factors that collaborate with PU.1 – the critical transcription factor initiating myeloid cell differentiation – to establish tissue-specific enhancers, ultimately defining the myeloid cell identity (72). This study indicates that microglia express high levels of *Cx3cr1* and *Nav3*, whereas peritoneal macrophages express *Lyz2*, *Fn1*, *H2-Eb1*, *Cebpb*, *Gata6*, and *Ciita* in a subset-specific manner. Whether brain-infiltrating macrophages have similar genetic profile to peritoneal ones is still to be determined; however, this study further demonstrates that microglia and peripheral macrophages have intrinsic genetic differences and propose the environment as a major determinant of these differences.

### Polarization markers

As mentioned above, after activation the commitment of microglia and macrophages can be either toxic or protective. Microglia can affect neuronal function and promote neurotoxicity (73) through the release of several pro-inflammatory molecules, such as IL-1β, TNF-α, proteases, and reactive oxygen species (ROS) (74). However, under certain circumstances microglia can be neuroprotective and promote adult neurogenesis and lesion repair (75, 76). Microglia can be neurosupportive through several mechanisms including glutamate uptake (77), removal of cell debris (78), and production of neurotrophic factors, such as insulin-like growth factor-1 (IGF-1) (79), glial cell-derived neurotrophic factor (GDNF) (80), and brain-derived neurotrophic factor (BDNF) (81). The dual nature of microglial polarization is similar to that of macrophages generally referred to as M1 or M2 (82). According to this classification, macrophages can develop a classic pro-inflammatory (M1) or an alternative anti-inflammatory (M2) polarization. Specific environmental signals induce these different polarization states (7). In particular, stimulation through toll-like receptor (TLR) ligands and INF-γ induces classical M1 activation, while IL-4/IL-13 stimulation favors the alternative M2 activation (83).

M1 and M2 canonical definitions represent the extreme of macrophage-polarized states documented *in vitro* (84). A generally accepted classification of the microglia/macrophage repertoire of polarized states indicates one M1 toxic polarization and three M2 polarization subtypes, M2a, M2b, and M2c, each with a specific function and pattern of marker expression (84). M1 is associated with phagocytosis, ability to kill pathogens by iron restriction, phagosome acidification, and ROS release (85–88). M1 phenotype markers include CD16, CD32, CD86, MHC II, and iNOS. M2a-polarized state is associated with immunity against parasites, recruitment of Th2 cells, tissue repair, and growth stimulation. This state is marked mainly by the expression of arginase-1, Ym1, and Fizz (85–95). M2b has either pro- or anti-inflammatory functions and is associated with memory immune response (B-cell class switch and recruitment of regulatory T cells). High IL-10 expression, MHC II, and co-stimulatory CD86 define the M2b pattern (85–87, 96–100). M2c is involved mainly in scavenging cell debris, has healing functions, and expresses arginase-1, CD163, and CD206 (85–87, 89, 90, 95, 100–102). An M0 phenotype has been recently introduced to indicate a non-polarized state. Adult microglia cultured with macrophage colony stimulating factor (MCSF) and TGF-β have a phenotype similar to fresh-cultured adult microglia and therefore have been defined to as M0 (71).

These antigenic fingerprints for polarization are well mirrored in some pathological conditions *in vivo*, including parasite infections, allergy, and cancer (83). In neurodegenerative disorders, myeloid cells often present mixed phenotypes indicating their plastic nature and their ability to acquire multiple phenotypes in response to environmental signals and time-dependent changes in the inflammatory milieu (83). As a further element of complexity, some markers are clearly assigned to a specific polarization state, while others do not clearly belong to one or the other. As an example MHCII and CD86 may be found in either the M1 or the M2 phenotype (84). MHCII is involved in antigen presentation to immune cells and CD86 functions as a co-stimulatory signal for T-cell activation. It is intriguing that microglia/macrophages acting as antigen-presenting cells are not easily classified as toxic or protective. Quite possibly T-cell activation needs to be finely tuned to yield a tolerization state, associated with protective action (103, 104), rather than an aggressive (auto)-immune response. For a satisfactory definition of myeloid cell polarization in CNS diseased states, further studies in models of pathology are needed. Events such as infiltration of leukocytes are important in orchestrating polarization, making the *in vivo* studies far more instructive than *in vitro* models, which cannot reproduce the complete network of recruited/activated cells.

### Reactive markers

The activation of microglia/macrophages is driven by many factors, including cytokines, chemokines, released degradation products, and extravasated molecules. Specific responses to these factors have been thoroughly reviewed elsewhere (105–108). Here, we provide information on the reactive markers that tag specific microglia/macrophage functions. In addition to the upregulation of constitutive molecules and the expression of polarization markers, activated microglia/macrophages also express novel antigens associated with specific functions. Reactive antigens include, among others, CD68, CD200, F4/80, CD14, HLADR, TLRs, heat shock protein (Hsp)-70, C3b/iC3b, CR3, and sodium calcium exchanger (NCX)1 (109–114). These markers indicate a reactive status, but they cannot be used to define whether myeloid cells have toxic or protective functions. The activation of microglia and macrophages is linked to different functions, including damage-sensing activity, chemotaxis, and phagocytosis. These events are among the most distinctive features of these cells, and specific markers, discussed below, apply to each reactive phase.

#### Damage-sensing activity

Microglia are distributed uniformly throughout the CNS. Specific microglial populations may exist in selective CNS regions (115), depending on the white or gray matter localization, proximity to vasculature, interaction with BBB components, biochemical features of the microenvironment, and substances released by neurons (116–118). Functionally, these adaptations guarantee tissue homeostasis under physiological conditions and are readily activated in case of threats to the CNS. Like macrophages, microglia are well equipped to sense any change in tissue homeostasis thanks to the expression of different surface receptors with damage-sensing functions. These receptors, generally referred to as pattern recognition receptors (PRR), bind ligands of different types: high mobility group box 1 (HMGB1), Hsp, hyaluronic acid and fibronectin produced by extracellular matrix degradation, apoptotic or injured cells, nucleic acids, immune complexes, mannose residues, and proteolytic enzymes (119). These ligands are called danger-associated molecular patterns (DAMPs) and are released as a consequence of insults to the CNS.

Toll-like receptors are a family of PRR that recognize a wide variety of danger signals and consequently activate different inflammatory cascades. These receptors, originally identified as initiators of innate immunity in response to exogenous microorganisms, have a role in the inflammatory response to ischemic injury in the absence of infection (120). TLRs are expressed by myeloid cells in response to activation and are involved in the switch toward M1 polarization (83) either by inducing NF-κB, which in turn induces pro-inflammatory cytokines (TNFα, IL-12, SOCS3) and HIF-1α to promote iNOS synthesis, or by inducing IRF-3, one of the main transcriptional regulators initiating M1 polarization (via STAT1) and M2 gene silencing (121). TLR4 has a pivotal role in the promotion of the M1 polarization (83). The induction of IRF-3 by brief activation of TLR4 or TLR9 can also have protective effects. Preconditioning with low doses of either the TLR4 ligand LPS or the TLR9 ligand CpG confers protection against ischemic injury through IRF-3 and IRF-7, and importantly, this effect is mediated by glial cells (122).

In the CNS, a few PRR are expressed by other cell types, such as NOD-like receptor proteins (NLRP) expressed in astrocytes (NLRP2) or neurons (NLRP1) and the-absent-in-melanoma-2 (AIM2)-like receptor, expressed in neurons. Along with caspases these receptors form the inflammasome and are involved in pyroptotic events (119). Non-surface-bound PRR exist too. These include the initiators of the complement cascade, such as serum proteins C1q, mannose-binding lectin (MBL), ficolins, and collectins, which circulate in the bloodstream and, on recognition of a wide array of target ligands expressed also by myeloid cells (123), trigger complement activation leading to injury progression (124).

Findings in animal models and in humans support the idea that there is a high degree of interaction between TLRs and complement. Complement receptors including C5aR, C3aR, and C5L2 crosstalk with TLRs, result in the regulation of innate immune and inflammatory responses in addition to regulation of adaptive immunity (125). This interaction may lead to the production of inflammatory molecules or dampening of excessive inflammation. The molecular mechanisms leading to this functional regulation are not completely clear but they include the possibility that complement receptor activation is needed for optimal TLR signaling or that complement fragments can directly activate TLRs (126). Crosstalk of complement components with other PRR is also described. C1q can inhibit NLRP3-dependent cleavage of caspase 3 and subsequent IL-1β cleavage, possibly by increasing the expression of negative regulators of the inflammasome activity, such as NLRP12 and/or POP1/ASC2 (127).

The complement system has a major role in the activation of microglia, cells that constitutively express the receptors for C1q and for cleavage products of C3, complement components that mediate phagocytosis and stimulate cytokine production by microglia. On binding, microglia become activated and in turn contribute to complement component production that feeds autocrine/paracrine signaling. Microglia respond to C1q with a marked shift toward pro-inflammatory activation in CNS diseases with BBB impairment (128). A role of C1q-mediated activation of microglia was identified in CNS development, when it is required to prune neuronal synapses (129). However, C1q influences a multiplicity of functions associated with macrophages, for instance upregulating IL-33, a cytokine that can amplify the M2 polarization induced by IL-13 (130, 131). Benoit et al reported that C1q induced the secretion of anti-inflammatory cytokines, such as type I interferon, IL-27, and IL-10 by human macrophages stimulated with low doses of LPS. These effects were abolished with higher doses of LPS, suggesting that this immunoregulatory activity occurs with limited inflammatory challenge (127).

There is also evidence suggesting the involvement of MBL, one of the activators of the lectin complement pathways, in the activation of microglia. MBL may indirectly induce the activation of microglia through enhancement of fibrin deposition (53), which drives microglial recruitment and activation (52).

The purinergic system mediates intimate crosstalk between microglia and neurons at synapses (132), based on extracellular ATP/ADP sensing (3). Microglia express P1 receptors – including all subclasses of adenosine receptors, e.g., adenosine A_1_, A_2_A, A_2_B, and A_3_ receptors (133) – and also P2 receptors, including ionotropic P2X (P2X_4_, P2X_7_) and metabotropic P2Y (P2Y_2_, P2Y_4_, P2Y_6_, P2Y_12_, and P2Y_14_) receptors (134–136). Microglia show a certain degree of constitutive expression of these receptors, allowing scanning activity during physiological surveillance. Surveying microglia express high levels of P2Y_12_ receptors (137) that are involved in branch extension. On activation, microglia can alter their set of purinergic receptors and respond to specific demands of the surrounding environment. Different purinergic receptors may be induced simultaneously to obtain cooperative effects, whereas others may function independently or be silent. For example, microglial activation and reactive transformation into their ameboid phagocytic phenotype require specific profiles of purinergic receptors: P2Y_12_ and A_3_ receptors cooperate to allow process extension (damage sensing), A_2_A receptors induce retraction (ameboid transformation), and P2Y_12_, P2X_4_, and A_1_ receptors interact to induce migration by ATP/ADP sensing (recruitment to lesion site). Finally, P2Y_6_ receptors sense UDP to start phagocytosis (138).

P2Y_12_ receptors have been proposed as pharmacological targets (137). As already mentioned, they take part in primary damage sensing through ADP-induced chemotaxis. Early inhibition of ADP sensing by ticagrelor induces protection from ischemia at 48 h and is associated with reduced microglia/macrophage recruitment to the lesion site and with decreased expression of pro-inflammatory mediators, such as iNOS, IL-1β, and MCP-1 (137). This suggests that microglia have an early toxic effect that can be counteracted by inhibiting their activation and homing to the lesion.

#### Chemotaxis

Following damage-sensing activity, microglia/macrophages become responsive to chemokines released by the host tissue and promote the invasion of the inflamed CNS site. The ability of infiltrating immune cells, such as macrophages, to migrate within the invaded tissue has long been known by immunologists. Macrophages abundantly express CC and CXC family surface receptors for chemokines, among which CCR2 plays a major role in driving macrophage invasion through monocyte chemoattractant protein 1 (MCP-1) sensing (66–68). Positivity to CCR2 can be exploited in mice to define a specific macrophagic subset, which expresses Ly6C, high levels of Gr1 and low levels of CX3CR1 (139). In humans, the homologous subset is CCR2^high^/CD14+/CX3CR1^low^. Macrophages belonging to this subset have been associated with the development of the M1 polarization state. A second subset, negative for CCR2, with a Ly6C-/Gr1^low^/CX3CR1+ phenotype, has its human homolog in CCR2^low^/CD14-/CX3CR1^high^ cells and is associated with the M2 final commitment (139). However, sharp commitment of CCR2 positive or negative subsets is not easily determined because high levels of its ligand MCP-1 (also known as CCL2) have also been described in a M2-polarized inflammatory environment (140).

Microglia express chemokine receptors of the CC and the CXC family that, with purinergic receptors, regulate the machinery for microglial movements. In the early metabolic crisis after acute brain injury, microglia may use chemotactic signals to extend/withdraw their branches so as to receive activatory stimuli and change morphology. Their dynamic proprieties have only been recently explored, using *in vivo* imaging by two-photon microscopy (3, 51). Microglia show constant movements of their branches to patrol the environment although microglial ability to displace within the inflamed tissue seems scarce. The fact that active microglia release a number of factors with paracrine effects including BDNF (141), IGF-1, and cytokines (142) implies that they need to reach their site of action. However, their ability to travel over brain tissue depends on the kind of danger signal received. In a model of spreading depression in organotypic slice cultures, causing synaptic activity impairment, microglia showed displacement by Lévy flight-like movements in response to diminished synaptic activity (143). In contrast, in models, such as TBI (induced by laser ablation) (3), photothrombotic stroke, or brain ischemia (3, 21, 40), microglia did not display any migratory behavior. Their lack of displacement after acute brain injury may be related to the rapid decline of ATP levels due to perfusion impairment, ATP being needed for the cytoskeletal modifications that microglia exploit for migration (3, 143).

Little is known about microglial dynamics at later times. Proliferating microglia might migrate and settle in specific lesioned areas where new ones are needed to re-establish tissue homeostasis. Further studies will help decipher microglia dynamics over time better, because the literature on this issue so far mainly focuses on acute points (few hours after insult).

Some markers may be used to detect microglia/macrophages responding to chemotactic stimuli. F4/80 is commonly used to stain these cells in tissue. Its expression is enhanced in recruited tissue macrophages compared to circulating monocytes, indicating that it is involved in adhesion and tissue migration. Microglia also express F4/80 at high levels after activation. Interestingly, the human homolog of F4/80, EMR1, is not present on mononuclear phagocytic cells, such as macrophages, and in human specimens, it preferentially stains eosinophils (144).

The NCX1 is involved in the calcium-mediated functions of microglia, including chemotaxis. Calcium influx through the action of the exchanger is a prerequisite for bradykinin-induced microglial motility (145). In a model of brain ischemia, microglia significantly upregulated NCX1, reaching a peak of expression 3 and 7 days after the insult. NCX1 expression was identified in round-shaped Iba1+ cells that progressively invaded the ischemic core. NCX1 knockout mice show impaired chemotaxis and microglia migration even in the heterozygous state. Impaired microglia chemotaxis in these mice dramatically increased the brain ischemic lesion, suggesting that NCX1 is needed to develop microglial protective functions (110).

#### Phagocytosis

Phagocytosis is a complex event, whose role in acute brain injury is still not clear. Phagocytic activity is not clearly linked to a specific M1 or M2 polarization state. Cells showing M1 toxic as well as M2c pro-healing polarization may have phagocytic functions (84), indicating that phagocytosis may serve either for killing cells (toxicity) or scavenging debris (protection).

There is *in vitro* evidence that M2-polarized cells enhance their phagocytic behavior (146). This is regarded as a protective function because phagocytosis is needed for removal of cell debris to limit the spread of toxic signals and potentially favor tissue healing. In organotypic slice cultures, microglia may be neuroprotective by engulfing neutrophils (147).

On the other hand viable neurons exposed to sub-lethal stimuli can also express “eat-me” signals that tag them for elimination (148), a process also referred to as primary phagocytosis or phagoptosis (149). Two key proteins involved in the elimination of viable neurons exposing phosphatidylserine have been identified, namely milk fat globule EGF-like factor-8 (MFG-E8) and Mer receptor tyrosine kinase (MerTK), both of which bridge phosphatidylserine-exposing neurons to vibronectin receptors on phagocytic cells (150–153). The fact that the lack of these proteins prevents neuronal loss and improves behavioral deficits demonstrates the detrimental function of primary phagocytosis.

In the CNS, myeloid cell phagocytic activity can also be labeled by CD68, or macrosialin, a scavenger receptor member of the lysosomal/endosomal-associated membrane glycoprotein (LAMP) family (8, 109), which is found mainly in phagocytic macrophages (154). *In vivo* observations suggest that the expression of CD68 is inversely related to that of M2 markers during the temporal evolution of the lesion (8) or in response to protective manipulations, such as LPS preconditioning (155), stem cell treatment (140, 156), and CX3CR1 deletion (40).

These observations confirm that the phagocytosis cannot be ascribed to either M1 or M2 and imply that, although phagocytosis is needed for limiting the propagation of danger signals, any excess has detrimental effects.

A major effector of phagocytosis is the complement system cascade (124, 157). All the complement activation pathways, including those activated by the danger signals released in response to acute brain injury, converge on the activation of C3 convertase that leads to C3 cleavage. Its products, C3b/iC3b, are specifically able to opsonize damaged or apoptotic cells and function as “eat-me” signals, driving phagocytosis. C3b/iC3b deposits are recognized by the CD11b/CD18 (or CR3) receptor and may be used to reveal neurons committed to die.

## Does Morphology Indicate Function?

Similar to phenotypic markers, morphological changes can provide information on microglia/macrophage functional commitment. A morphological description is thus a priority to clarify the specific functional significance of these cells in brain pathology. The literature has explored the morphology of myeloid cells in physiological or diseased conditions with the aim of defining specific shape indicators of a given active/functional state (40, 57, 158). This can be done by combining the morphological data from imaging with those from conventional molecular analysis – immunostaining, real time PCR, western blots – in different experimental settings.

Microglia/macrophage activation is usually measured by immunohistochemical assessment of the specific antigenic markers discussed above, but this does not take into consideration morphological changes and shape descriptors. Actin polymerization leading to microglia/macrophage shape changes is strongly related to ATP/ADP availability and morphological changes are therefore finely tuned by the injured microenvironment.

**Figure 3:**
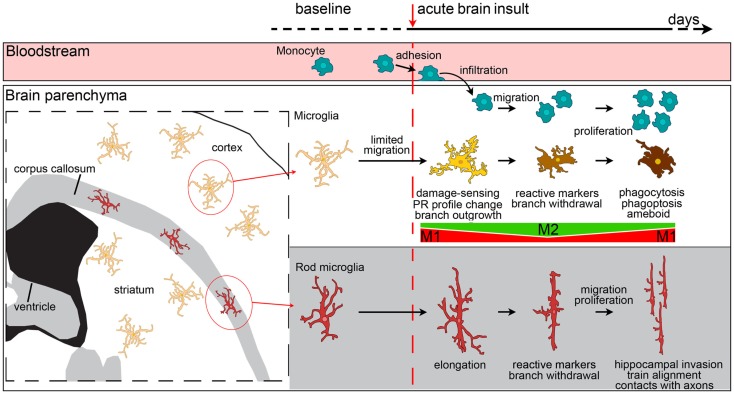
**Evolution of myeloid cell morphology and phenotype in the brain during the acute phases of brain injury**. Under physiological conditions, monocytes, microglia and rod microglia populate specific compartments, respectively blood, gray matter and white matter. After an insult to the brain, monocytes, microglia, and rod microglia are activated. Monocytes migrate to lesioned areas, whereas gray matter microglia are less likely to displace. Microglia change morphology in a time-dependent fashion, sprouting new ramifications soon after injury, and then withdrawing branches to develop an ameboid phenotype. Both infiltrated monocytes and microglia change their phenotypical profile, acquire specific functional commitments, and proliferate with time. In the very early phases after injury, microglia have a M1 phenotype, then, with the recruitment of macrophages, both myeloid populations upregulate M2 markers. The peak of M2 marker expression soon vanishes and is followed by upregulation of M1 markers that lasts longer. *In vivo*, mixed polarization phenotypes are observed and the M1-M2 definition may only serve to set the extremes of a continuum of polarization states. Macrophages from blood-borne monocytes seem to be more able to express M2 markers than microglia. In the white matter, rod microglia activate after injury by enhancing their elongated morphology, expressing reactive markers (CD68, MHCII), and migrating to other areas, such as the hippocampus. Rod microglia also cluster into trains of cells that align to neuronal axons. Neither the exact phenotype profile nor the functions of activated rod microglia are fully understood, though a role of these cells in synaptic stripping due to their contacts with axons has been hypothesized.

Microglia show a wide range of morphological transformations, from highly ramified with thin branches, to hypertrophic and ameboid with no branchings. In between these extremes, they can acquire a variety of shapes that mirror their functional state [Figure 3 (57)]. Macrophages, in contrast, are less prone to morphological changes. However, a certain amount of shape change is possible for macrophages too, because due to their plastic nature they can change their contractility state and establish interactions with the extracellular matrix and with cell surface adhesion molecules (159). When macrophages are cultured on substrates that force their elongation, such as arrays of narrow fibronectin lines, they start upregulating the M2 markers arginase-1, CD206, and Ym1 and reduce the release of pro-inflammatory cytokines (160). The *in vitro* induction of M2 phenotype by IL-4/IL-13 treatment causes elongation of the cultured macrophages, indicating that this is needed to drive the M2 polarization and, more generally, that geometry plays a role in the phenotype definition (160). Whether elongation defines polarization *in vivo* needs investigating because infiltrating cells may elongate merely as a consequence of crawling along vessel walls or a perivascular location.

Elongation seems to be an important feature for the definition of microglial functions as well. The white matter is populated by a specific microglial subtype, which has a bipolar, elongated morphology, called rod microglia [Figure 3 (161)]. These cells are close to neurons (162, 163) and take part in axonal damage and recovery after injury (118). In other brain areas, elongation may result as a consequence of directional ramification extending toward a specific site of injury. This process may be more evident at early times after injury, being necessary during the damage-sensing microglia re-orientation. As early as 24 h after ischemic injury, microglia are not elongated and display a reactive hypertrophic ameboid morphology with numerous short processes, symmetrically extending from the cell soma (40). This morphological state correlates with increased lysosomal activity and, interestingly, is decreased in CX3CR1-deficient mice that show increased expression of M2 markers and concomitant protection from ischemic injury (40). A protective role for ramified microglia was suggested by Vinet et al. who showed that these microglia not only survey their microenvironment but also contribute to protecting neurons during neurodegeneration induced by the glutamate receptor-agonist *N*-methyl-d-aspartic acid (158).

**Figure 4:**
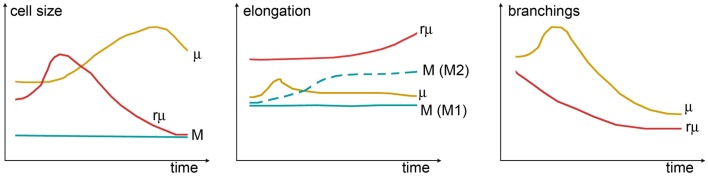
**Shape changes of different myeloid cell types over time**. Cell size (left panel) can be measured by assessing cell area or cell width (caliper). While in monocytes (M), the size is constant, microglia (μ) grow larger (hypertrophic reactive morphology), and rod microglia (rμ) first grow and then shrink (elongated not ramified morphology). Elongation (center panel) can be evaluated from the ratio between the cell’s major and minor axes. A certain degree of elongation is selectively associated with M switch to M2 polarization. Elongation of μ might occur during early damage-sensing as μ direct ramifications toward the site of injury. Then, along with ameboid transformation, μ recover their symmetric shape. In contrast, rμ progressively elongates so as to align with axons. The rate of branching (right panel) can be measured by counting the number of ramifications or by Sholl analysis. M does not have ramifications, while μ or rμ withdraw branchings over time to acquire, respectively, the ameboid or the elongated morphology: μ show a transient increase of ramifications early after injury that may be related to damage-sensing activity. Shape changes have been plotted according to the data and observations described in Ref. (40, 118, 160, 161, 164, 165).

The complex morphological features of specific myeloid populations can be evaluated by measuring different shape indices, whose temporal variations are clearly indicative of functional states, providing a quantitative analysis of shape changes (Figure 4). As an example, the longest branches of surveying microglia are found within a distance of 20–25 μm from cell centroid when measured by Sholl analysis *in vivo* in mice (40). One hour after transient ischemia, the longest branches are found at 30–35 μm from cell centroid indicating ramification sprouting, a process involved in the sensing of danger signals. Other changes of myeloid cells after transient ischemia are associated with cell size. One day after transient ischemia, CD11b+ cells display a mean cell area of 85.8 μm^2^, far higher than that in naïve mice (54.6 μm^2^). Similarly, myeloid cells increase perimeter (75.3 μm) and cell caliper (20.1 μm) after ischemia compared to controls (61.5 and 17.1 μm, respectively) (41).

Microglial morphology at late times after acute injury or in conditions of chronic activation have been explored little. After TBI, microglia develop a primed phenotype, defined by increased Iba1 labeling and hypertrophic cell soma that persists up to 30 days after insult (166). Primed microglia respond to a secondary inflammatory challenge with quick activation, yielding depressive-like behavior.

As amply discussed, microglia modify their shape dynamically because they can extend or withdraw their branches. While the number and length of ramifications are indicative of morphological features associated with microglial function, it is not clear whether the rate of extension/withdrawal per ramification reveals a given state. The average speed of extension/retraction (micrometers per minute) declines during a vascular occlusion when ATP is not available, but soon recovers to the baseline values when new perfusion is allowed (21). This implies that after ischemia the intrinsic process dynamics of the activated microglia remain unaltered. Results are similar with LPS because the process motility does not change either 2 or 28 days after LPS (167). Hypothetically microglia may quickly rearrange the length and number of branches and change morphology without losing their exploratory ability. However, the residual availability of an energy source is important for their movements, so environmental changes can alter microglial exploratory behavior under specific metabolic circumstances. This point calls for further studies to understand the dynamic response of microglia during their morphological transformation.

## Functional Consequences of Chronically Activated Microglia

To ensure brain homeostasis, microglia need to finely tune their physiological activity. The key to this control is the neuron-mediated inhibitory activity which, under normal conditions, prevents microglia from becoming active (6). Neurons control microglia through several components, including contact-dependent inhibitory signals, such as CD200-CD200R (168), Hsp60–TREM2–DAP12 (169), CD22-CD45 (170), and CD47-CD172 (171), and soluble molecules such as ICAM-5 (172), fractalkine (CX3CL1) (173), IL-10 (174), and TGF-β (175). Any change in microglial activity control may lead to chronic activation or a primed state with specific functional consequences. Here, we discuss three cases in which microglia are chronically activated and their implications.

### CX3CR1-deficient microglia acquire a chronic active state

CX3CR1, selectively expressed in the brain by microglia, is an important regulator of their activity. The main effect of CX3CR1 binding to its unique ligand CX3CL1 released by neurons is to control microglial activation. Under physiological conditions, the neuronal membrane-anchored CX3CL1 is cleaved by the disintegrin-like metalloproteinases ADAM10 and ADAM17 (176) and is released in its soluble form. Continuously released CX3CL1 keeps microglia in a non-reactive state. The generation of *cx3cr1*^GFP/GFP^ mice, knockout for CX3CR1 and referred to here as *cx3cr1^-/-^* mice, has led to a number of studies investigating CX3CL1:CX3CR1 signaling either under physiological conditions (177) or in CNS diseases (178–181), showing the involvement of this pathway in several aspects of development, homeostasis and injury, including synaptic pruning, promotion of neuron survival, synaptic transmission and plasticity, enhancement of synaptic networking and facilitation of neuropathic pain circuits (173).

In *cx3cr1^-/-^* mice, the suppressive function of CX3CL1 is prevented and this causes a chronic microglial activation state, with a larger number of branches than in WT mice, as shown by *in vivo* two-photon microscopy (40). This state has been associated with less hippocampal neurogenesis and cognitive deficits (177, 182, 183).

After an acute insult, such as brain ischemia, affected neurons significantly reduce the release of CX3CL1, allowing microglial activation (184, 185). Chronically activated microglia in *cx3cr1^-/-^* mice respond to acute injury by enhancing their ramified morphology, downregulating the phagocytic activity and acquiring features of M2 polarization, together with protection from ischemic injury (40). There are also reports of early protective effects of CX3CR1 deletion in different models of acute brain injury, including ischemia, trauma, or spinal cord injury (SCI) (179, 181, 186, 187). However, this response may be transient and at longer times other events may take place, changing the overall impact of CX3CR1 deficiency. Recruitment of the CX3CR1+/Ly6C^low^/CCR2- macrophage subset has been associated with beneficial effects as this population is capable of tissue healing (188). CX3CR1 deficiency would impair the recruitment of this protective population, resulting in a detrimental phenotype.

The deleterious consequences of CX3CR1 deficiency at late stages of pathology have been described in ischemia and SCI (189, 190). There is ample literature on *cx3cr1^-/-^* mice in chronic CNS diseases (67). In models of lateral amyotrophic sclerosis (178), Parkinson’s disease (PS) (191) and Alzheimer’s disease (192), the absence of CX3CR1 has been associated with a worse outcome possibly due to the chronic pro-inflammatory function of CX3CR1-deficient microglia (193, 194).

These findings indicate that specific microglia signaling pathways may give opposite outcomes depending on the temporal pattern of their action or on the disease stage. The longitudinal effects of CX3CR1 deficiency at different stages of pathology (Table 1) need to be carefully investigated to understand the exact role of the CX3CL1:CX3CR1 axis in brain injury.

**Table 1:** **Effects of CX3CR1 deficiency in different models of CNS diseases**.

Model	Time from injury	Deletion protective?	Effects of deletion	Reference
SCI	5–35 days	Yes	↑ hindlimb function, ↓ myelin and axon loss, ↑ CD45, ↓ IL-6, and iNOS	(181)
	42 days	No	Worsened locomotor function, ↓ myelin, ↑ infiltrating monocytes/macrophages	(189)
pMCAo	24 h	Yes	↓ ischemic volume	(187)
tMCAo	3 days	Yes	↓ infarct size	(186)
	1–3 days	Yes	↓ infarct size, ↓BBB breakdown, ↓ apoptosis, ↓microglia, and ↓IL-1β	(179)
	24 h	Yes	↓ infarct size, ↑ ramification in microglia, ↓ CD11b and CD68, ↑ CD45^high^, ↑Ym1, and ↓iNOS	(40)
	72 h	Yes	↓ infarct size, ↓ neurological deficit, ↓ apoptosis, ↓ IBA1, and CD45^high^, ↑ Ym1, ↓ iNOS, ↓ microglia proliferation, ↓ ROS, ↓ IL-1, IL-6, and TNF-α	(195)
	43–60 days	No	↑ microglia activation, ↑ IL-1β, and TNF-α, ↓ IL-4 and IL-10, worsens cognitive functions	(190)
LPS	4 days	No	↑ TUNEL, ↑ IL-1β	(178)
PD	7 days	No	↑ loss Nissl-stained cells, ↑ loss TH-IR	(178)
ALS	15–20 weeks	No	↓ neuronal cell density, ↓ motor function, ↓ survival	(178)
AD	28 days	No	↑ IL-6 and TNF-α, ↑Mac2 (activated microglia), ↓ cognitive and memory deficits	(192)
	28 days	Yes	↓ neuronal loss	(196)
	28 days	Yes	↓ Aβ deposition, = CD45, ↓GFAP, ↓ TNF-α and MCP-1/CCL2, ↑IL-1b, microglia more rounded, ↓ CD68, ↑ Aβ phagocytosis	(197)
	28 days	Yes	↓ Aβ deposition, ↑ Aβ phagocytosis, ↑ microglia proliferation, = neuronal injury	(198)
Tau pathology		No	↑ MAP phosphorylation, behavioral impairments, ↑ microglial activation, ↑IL-1b	(199)

*The role of the fractalkine receptor (CX3CR1) in CNS pathologies has been studied using cx3cr1^-/-^ mice. In different disease models, including SCI, permanent ischemia (pMCAo), transient ischemia (pMCAo), lipopolysaccharide (LPS)-induced inflammation, Parkinson’s disease (PD), amyotrophic lateral sclerosis (ALS), Alzheimer’s disease (AD), and Tau pathology. The results are often contrasting and depict a puzzling situation. A potential general hypothesis is that CX3CR1 deletion is protective in the early phases after acute injury, but becomes deleterious at later stages or in chronic conditions*.

### Microglia change their morphology and dynamics in the aging brain

In aged mice (27–28 months), microglia increase their cell soma volume and shorten mean ramifications. This is accompanied by quicker soma movements and less baseline process motility than in young mice [3 months (200)]. Although the morphological features of microglia in aging brains are reminiscent of a reactive state, it is not clear whether the aged microglia are truly activated. It is likely that these cells enter a dysfunctional state that may affect the integrity of other structures with which they come into contact, such as neuronal or vascular networks. Possible consequences of the dysfunctional behavior of aged microglia include dysregulated response to injuries, changes in neuroprotective functions and increased toxic responses (200).

Microglial modifications during aging may be related to changes in either central or peripheral inflammatory events over the lifespan. As already discussed, many molecules with paracrine effect are needed for controlling microglial activation under physiological conditions. However, aging may change the expression of these molecules, resulting in changes in microglial activation states. An interesting example of dysregulated microglia control involves TGFβ1. This cytokine has been defined as a determinant of microglia support of CNS homeostasis, favoring the microglia anti-inflammatory phenotype through IRF-7 signaling (71). However, chronic exposure to TGFβ1 has the opposite effect, inhibiting IRF-7 and consequently impairing the anti-inflammatory switch of microglia (201). This latter effect may be important during aging because this condition has been reported to induce chronic TGFβ1 upregulation (202) and can thus cause chronic pro-inflammatory microglial activation.

As for peripheral inflammatory changes, recent work demonstrates that mice injected systemically with blood from aged mice have decreases in neurogenesis, learning, and memory – all effects mediated by the chemokine system (203). The rules that govern the interaction between systemic inflammation and microglial behavioral changes throughout life are far from understood. However, a recent hypothesis attributed to a lifetime peripheral inflammatory state a fundamental role in degenerative pathologies, possibly through an impact on central inflammation.

The upregulation of MCP-1, a chemoattractant molecule for myeloid cells, in multiple models of neurodegenerative diseases, including Alzheimer’s, Parkinson’s, amyotrophic lateral sclerosis, and prion disease (204–207), has been linked to the possibility that myeloid cell lineages play a major pathophysiological role. In chronic disease states, microglia appear to be primed by earlier pathology or genetic predisposition and respond with hyperactivation to inflammatory stimulation. This might lead to an adaptive CNS inflammatory response changing into one with deleterious outcomes (164). Other authors reported a major involvement of peripheral macrophages rather than microglia in driving chronic diseased states. In a model of Parkinson’s disease induced by injection of 1-methyl-4-phenyl-1,2,3,6-tetrahydropyridine (MPTP), peripheral M1 macrophages activation proceeds by days microglia activation and neuronal loss (208). This suggests that, at least in this model, innate peripheral inflammation is a critical player rather than a mere consequence.

### Microglia in depressive behavior

Individuals who suffered an acute brain injury have a higher incidence of depressive behavior (209). The etiology of increased depression is still not clear but it may be inflammatory related. Clinical and experimental data indicate a cause/effect relationship between inflammation and depression (210), which is mediated, at least in part, by high levels of circulating inflammatory cytokines (211). Experimental studies suggested a strong correlation between the volume of damaged brain tissue and the extent of systemic immune alterations, irrespective of the site of infarct (212). Patients with acute brain injury have high levels of circulating IL-6, and antidepressant therapies fail to reduce TNF-α (211). These cytokines are released soon after acute brain injuries and accumulate in cerebrospinal fluid and serum (213–215). Moreover, markers of neuroinflammation (e.g., CD68, CR3/43) persist in the brain parenchyma up to 16 years after acute CNS insults (216).

Recent work on experimental models helps attribute a specific role to microglia in driving neuroinflammation-induced depressive behavior. Fenn et al. reported that experimental TBI induces microglia priming that results in a hyperinflammatory response to an immune challenge, persisting weeks to months after injury, finally triggering the development of depressive-like behavior (166).

It is likely that increased microglial sensitivity to secondary inflammatory challenges (e.g., stressors, infection and injury) involves mediators of the complement system and PRRs expressed on the microglial cell surface. However, this has so far been studied little, and the exact role of primed microglia in late stages after initial brain insult remains unexplored.

## Stem Cell Treatment as a Proof of Principle of the Possibility of Driving the Microglia Phenotype for Therapeutic Purposes

Promoting a microglial neuroprotective phenotype is an emerging therapeutic goal for CNS conditions. One strategy of interest is the use of stem cells. There is increasing evidence of the efficacy of mesenchymal stromal cells (MSC) in acute brain injury (217). The paracrine effects of MSC-released bioactive factors modify the injured microenvironment favoring reparative and restorative processes. Among the cerebral populations affected by MSC, microglia are an important target. *In vitro* and *in vivo* evidences show that MSC can tune protective microglial polarization. A direct link between MSC infusion and changes in the polarization of microglia has been documented *in vitro*, exposing MSC/microglia co-culture to different toxic stimuli (140, 146, 218). Primary murine microglia co-cultured with MSC increase the mRNA and protein expression of the M2 markers Ym1 and CD206 (140). This is obtained when MSC/microglia are co-cultured in direct contact or in a transwell system, the latter allowing only a paracrine effect. More interestingly, MSC may reverse the M1 phenotype acquired by microglia after a pro-inflammatory challenge (by either TNFα + IL-17 or TNFα + IFN-γ stimulus), inducing M2 pro-regenerative traits, as indicated by the downregulation of iNOS and upregulation of Ym1 and CD206 mRNA expression (140). Results are similar after microglia exposure to a LPS toxic stimulus and MSC co-culture. Again, in control and inflamed conditions, MSC upregulate mRNA and protein levels of the M2 marker arginase-1 (218) and can to reverse the microglial M1 phenotype increasing the expression of the M2 marker CD200R (146).

Besides the expression of specific markers, the M2 phenotype is associated with a specific profile of released cytokines. MSC may alter the ratio of IL-10 to TNF-α in favor of the anti-inflammatory cytokine IL-10 in the supernatants of LPS-stimulated microglia cultures (218). In addition, the secretome obtained even with unchallenged MSC reduces the production of TNFα, IL-1β, RANTES/CCL5, and MIP-2 by exposed microglia (219). Interpretation of the microglia cytokine profile after MSC/secretome exposure is not always easy. Rahmat et al. showed that together with a decrease in the production of TNF-α, MSC increase the production of IL-6, a cytokine usually considered pro-inflammatory (220). Kim and Hematti found a similar pattern (221) after MSC/macrophage co-culture, revealing a new CD206+ M2 subtype with a IL-10^high^/IL-12^low^/IL-6^high^ phenotype and low secretion of TNF-α^low^, potentially implicated in tissue repair. Increases in MCP-1/CCL2 and IL-1β are reported in MSC/microglia co-culture (146, 218). MCP-1/CCL2 is a potent chemoattractant for monocytes and may have a role in tissue repair and regeneration (222).

Although several aspects of MSC/microglia interaction are not completely clear yet, there are good reasons to use MSC *in vivo* as a driver of microglia post-conditioning after brain injury with the aim of pushing the M2 protecting phenotype. The first demonstration of MSC’s ability to induce *in vivo* M2 protective modulation after acute brain injury was provided by Ohtaki et al. (223) after stroke in mice. After 15 min transient, common carotid artery occlusion followed by transplantation of 100,000 human MSC into the dentate gyrus the mice had improved neurological function and less neuronal cell death in the hippocampus, with increased protein expression of M2 markers (Ym1, IGF-1, and Gal-3). Microarray assays indicate that MSC downregulate more than 10% of the ischemia-induced genes, most of them involved in inflammatory and immune responses, showing that cell treatment has a potent immunomodulatory effect. M2 induction after MSC transplantation has also been seen after SCI (224) or TBI (156), and in both cases M2 polarization is associated with less scar tissue formation and improvement of behavioral deficits.

Our group has provided a detailed description of the effects on microglia/macrophage after MSC infusion in TBI mice, associating MSC-induced protection with increased activation of microglia/macrophages (156). This indicates that MSC *in vivo* do not reduce the inflammatory response, but rather pushes it toward a more protective phenotype. MSC enhance the expression of M2 markers (Ym1, Arg1, and CD206) over time, making the M2 phenotype last longer (up to 7 days) than in untreated animals (3 days). MSC induce general reprograming of the microenvironment, including increases of IL-10 and VEGF, reduction of astrogliosis and induction of the axonal regeneration marker GAP-43. These data indicate that the beneficial traits of microglia/macrophages induced by MSC skew the balance of the immune response toward protection and regenerative processes, further confirming that acting on the microglia/macrophage modulation would offer therapeutic benefit.

## Conclusion

Recent evidence has helped define a new role for brain immune cells, highlighting their involvement in several stages of development, homeostasis, aging, or response to injury. Microglia and macrophages are the main players in neuroinflammation, and in view of their plastic nature, they may develop either toxic or protective functions. Specific commitments of these cells are closely associated with microenvironment signals, including metabolic crisis, exogenous/endogenous danger-associated molecules, and neuron-mediated activity control. Research in experimental settings has begun to lay a basis for understanding the behavior of microglia and macrophages in different physiological or pathological conditions, offering reasons for their therapeutic use. However, many aspects of their function, e.g., long-term response, crosstalk with other brain cells, and selective roles of microglia vs. macrophages, still need to be properly investigated if we are to acquire the ability to switch neuroinflammation protective functions.

## Author Contributions

MGDS, EZ, and CP drafted the manuscript; SF and FP drafted the manuscript and prepared the figures.

## Conflict of Interest Statement

The authors declare that the research was conducted in the absence of any commercial or financial relationships that could be construed as a potential conflict of interest.

## Abbreviations

AD, Alzehimer’s disease; ADAM, a disintegrin and metalloproteinase domain-containing protein; ADP, adenosine diphosphate; AIM2, absent in melanoma 2; ASC2, apoptosis-associated speck-like protein containing a CARD 2; ATP, adenosine triphosphate; BBB, blood–brain barrier; BDNF, brain-derived neurotrophic factor; CC, corpus callosum; CNS, central nervous system; CX, cortex; DAMPs, danger-associated molecular patterns; DAP12; DNAX activation protein of 12kDa; EMR1, EGF-like module-containing mucin-like hormone receptor-like 1; GDNF, glial cell-derived neurotrophic factor; GFP, green fluorescent protein; HIF-1α, hypoxia-inducible factor-1α; HMGB1, high mobility group box 1; Hsp, heat shock protein; Iba1; ionized calcium-binding adaptor 1; ICAM; intercellular adhesion molecule; IFN-γ, interferon-γ; IGF-1, insulin-like growth factor-1; IL, interleukin; iNOS, inducible nitric oxide synthase; LAMP; lysosomal/endosomal-associated membrane glycoprotein; LPS, lipopolysaccharide; LV, lateral ventricle; μ, microglia; M, macrophage; MBL, mannose-binding lectin; MCA, middle cerebral artery; MCP-1, monocyte chemoattractant protein 1 (also known as CCL2); MCSF, macrophage colony stimulating factor (MCSF); MerTK, Mer receptor tyrosine kinase; MFG-E8, milk fat globule EGF-like factor-8; MHC, major histocompatibility complex; MSC, mesenchymal stem cells; NCX, sodium calcium exchanger; NF-κB, nuclear factor kappa-light-chain-enhancer of activated B cells; NLRP, NOD-like receptor protein; NO, nitric oxide; OGD, oxygen-glucose deprivation; PCR, polymerase chain reaction; PD, Parkinson’s disease; POP1, pyrin-only protein 1; PRR, pattern recognition receptor; PTP, protein tyrosine phosphatase; RANTES, regulated upon activation normal T cell expressed and presumably secreted; rμ, rod microglia; ROS, reactive oxygen species; SCI, spinal cord injury; STR, striatum; TBI, traumatic brain injury; TLR, toll-like receptor; TNFα, tumor necrosis factor α; TGF-β, transforming growth factor β; TREM2, triggering receptor expressed on myeloid cells 2; WBC, white blood cells; WT, wild type.
